# Coordinate cis-[Cr(C_2_O4)(pm)(OH_2_)2]^+^ Cation as Molecular Biosensor of Pyruvate's Protective Activity Against Hydrogen Peroxide Mediated Cytotoxity

**DOI:** 10.3390/s8084487

**Published:** 2008-08-01

**Authors:** Dagmara Jacewicz, Michał Szkatuła, Agnieszka Chylewska, Aleksandra Dąbrowska, Michał Woźniak, Lech Chmurzyński

**Affiliations:** 1 Department of General and Inorganic Chemistry, University of Gdańsk, Sobieskiego 18/19, 80-952 Gdańsk, Poland; E-mails: dagmara@chem.univ.gda.pl (D. J.); lapa@chem.univ.gda.pl (A. Ch.); alex@chem.univ.gda.pl (A. D.); 2 Department of Medical Chemistry, Medical University of Gdańsk, Dębinki 1, 80-211, Gdańsk, Poland; E-mails: mszkatula@amg.gda.pl (M. Sz.); mwozniak@amg.gda.pl (M. W.)

**Keywords:** carbon dioxide, molecular biosensor, oxidative stress, hydrogen peroxide

## Abstract

In this paper instrumental methods of carbon dioxide (CO_2_) detection in biological material were compared. Using *cis*-[Cr(C_2_O_4_)(pm)(OH_2_)_2_]^+^ cation as a specific molecular biosensor and the stopped-flow technique the concentrations of CO_2_ released from the cell culture medium as one of final products of pyruvate decomposition caused by hydrogen peroxide were determined. To prove the usefulness of our method of CO_2_ assessment in the case of biological samples we investigated protective properties of exogenous pyruvate in cultured *osteosarcoma 143B* cells exposed to 1 mM hydrogen peroxide (H_2_O_2_) added directly to culture medium. Pyruvic acid is well known scavenger of H_2_O_2_ and, moreover, a molecule which is recognized as one of the major mediator of oxidative stress detected in many diseases and pathological situations like ischemia-reperfusion states. The pyruvate's antioxidant activity is described as its rapid reaction with H_2_O_2_, which causes nonenzymatic decarboxylation of pyruvate and releases of CO_2_, water and acetate as final products. In this work for the first time we have correlated the concentration of CO_2_ dissolved in culture medium with pyruvate's oxidant-scavenging abilities. Moreover, the kinetics of the reaction between aqueous solution of CO_2_ and coordinate ion, *cis*-[Cr(C_2_O_4_)(pm)(OH_2_)_2_]^+^ was analysed. The results obtained enabled determination of the number of steps of the reaction studied. Based on the kinetic equations, rate constants were determined for each step.

## 1. Introduction

Oxygen-derived free radical anion formed by mono-, di- or trivalent reduction of molecular oxygen have been involved in many disease such as diabetes, hypertension, ischemia-reperfusion injury, neurodegenerative disorders, atherosclerosis and others [1-4]. The presence of reactive oxygen species (ROS) including: superoxide anion (O_2_^·-^), hydrogen peroxide (H_2_O_2_) and more potent oxidant hydroxyl radical (HO^·^) can lead to irreversible damages of cell's components - proteins, lipids and DNA [5-7]. Since mammalian cells even in physiological state are constantly exposed to free radicals, they require a functional system of antioxidants to protect themselves from the toxic actions of ROS [8]. The most characteristic elements of this system are superoxide dismutase (SOD), catalase, peroxiredoxins, α-tocopherol, glutathione and ascorbic acid [9]. Among others also pyruvic acid belongs to this battery of anti-oxidants and it seems to be useful and powerful scavenger of hydrogen peroxide and other peroxides.

H_2_O_2_ is considered as a one of the most important mediators of oxidative stress. It can be produced intracellularly, especially by respiratory chain reaction and by extracellular oxidative burst mechanism used by activated inflammatory cells. In the presence of reduced transition metals, H_2_O_2_ can be transformed to more aggressive hydroxyl radical, which at least partially contributes to the cytotoxity of H_2_O_2_ [10]. Moreover, the ability of H_2_O_2_ to penetrate biological membranes allows to affect not only cells in which it was produced but also neighbouring cells [9]. The excessive production of H_2_O_2_ was noted in aging brain, in ischemia-reperfusion situations and the increase the level of H_2_O_2_ probably participates in the neuronal damage observed in Parkinson's disease [11-13]. Taking into consideration all those facts it seems that antioxidant properties of pyruvic acid (CH_3_COCOOH), which is also recognised as compound involved in energy production can play an important protective role against the toxity of hydrogen peroxide [14-16]. Furthermore, the pyruvate can be considered as an endogenous, as well as a specific exogenous antioxidant since many types of cells including neurons release into plasma and serum where it can protect them against the acting of H_2_O_2_ [17]. According to Mallet RT et. all for the antioxidant properties of pyruvic acid are responsible its chemical structure and the patterns of its cellular metabolism [18]. The α-ketocarboxylate structure enables pyruvate to detoxify H_2_O_2_ in a direct, nonenzymatic reaction in which H_2_O_2_ is reduced to H_2_O and pyruvic acid undergoes transformation to CO_2_ and acetate [19].

(1) $$\text{pyruvate}+{\text{H}}_{2}{\text{O}}_{2}\to \text{acetate}+{\text{CO}}_{2}+{\text{H}}_{2}\text{O}$$

Despite the fact that the above presented reaction was described in 1904 by Holleman the mechanism of the antioxidant action of the pyruvate has not been fully elucidated to date. In the present study, using a novel specific molecular biosensor - coordinate ion, *cis*-[Cr(C_2_O_4_)(pm)(OH_2_)_2_]^+^, where pm denotes pyridoxamine and stopped-flow spetrophotometry method we were able to correlate the amount of liberated CO_2_ as one of final products of the chemical reaction between exogenous pyruvate and exogenous H_2_O_2_ with the cytoprotective activity of pyruvate. We also analyzed kinetics of this reaction using both biological and chemical models. Moreover, it was proved that reactive oxygen species could modulate activity of mitochondrial enzyme pyruvate dehydrogenase (PDH) [20]. It has been although found that no change in PDH activity in the brain occurred following ischemia but as much as 65% inhibition following 24h of reperfusion and H_2_O_2_ generation was detected [21]. This is why we decided to measure the protection of pyruvate, as well as CO_2_ level after 24 hours of H_2_O_2_ treatment. Thus it seems highly probable that nonenzymatic activity between pyruvate and H_2_O_2_ can substitute deficiency of PDH activity.

**Figure 1.**
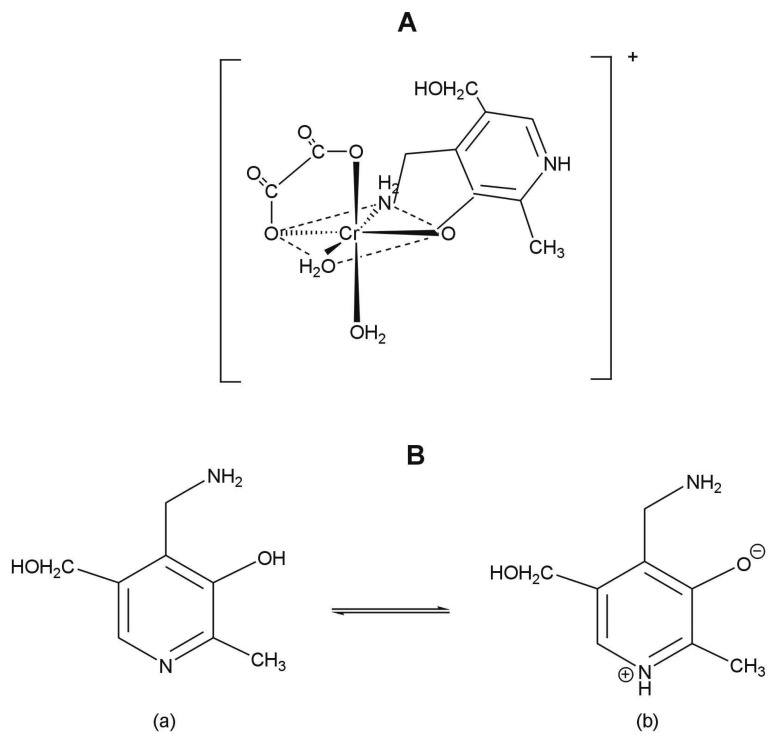
**(A)** Structure of the synthesized coordination compound of Cr(III); **(B)** Tautomers of pyridoxamine.

The structure of the synthesised complex of chromium(III) is shown in Figure 1 (A). It can be seen that the metal ion is coordinated by two oxygen atoms from oxalato anion, two oxygen atoms from two aqua ligands and one oxygen and one nitrogen atoms from pyridoxamine in *cis*-[Cr(C_2_O_4_)(pm)(OH_2_)_2_]^+^ ion.

The pyridoxamine acts as a bidentate ligand coordinating through the deprotonated phenolic-oxygen and the nitrogen donor atom from the amine group. Pyridinium nitrogen is protonated and it does not participate in binding of coordination center. For pyridoxamine, two types of tautomer forms are possible, as shown in Figure 1 (B). The structure of the ligand in pyridoxamine corresponds to type b. This structure was proposed also on the basis of the already published solution studies [22-25].

The selection of chromium(III) as the coordination center allows to obtain inert complexes undergoing slow transformations at ambient temperature, thus enabling investigation of the kinetics and mechanism of the processes under favorrable conditions. Furthermore, studies on the kinetics and mechanism of CO_2_ uptake by the coordination compounds consisting of the inert Cr(III) ion and a ligand molecule of pyridoxamine are aimed at elucidation of the mechanism of action of one of the metabolic steps. Carbon dioxide is caught by complexes of transition metal ions from biological material stoichiometrically as carbonate anion. This anion can be removed in the presence of acid as carbon dioxide (hydrolysis - reverse reaction to uptake) stoichiometrically too. The course of these two reactions can be presented as follows:

(2) $${\text{CO}}_{2}+[{\text{M}}_{\text{trans}}]\overset{\text{uptake}}{\underset{\begin{array}{c} +{\text{H}}^{+}\\ \text{hydrolysis} \end{array}}{⇌}}{\left[{\text{M}}_{\text{trans}}({\text{O}}_{2}\text{CO})\right]}^{\text{n}}$$

## 2. Results and Disscusion

**Figure 2.**
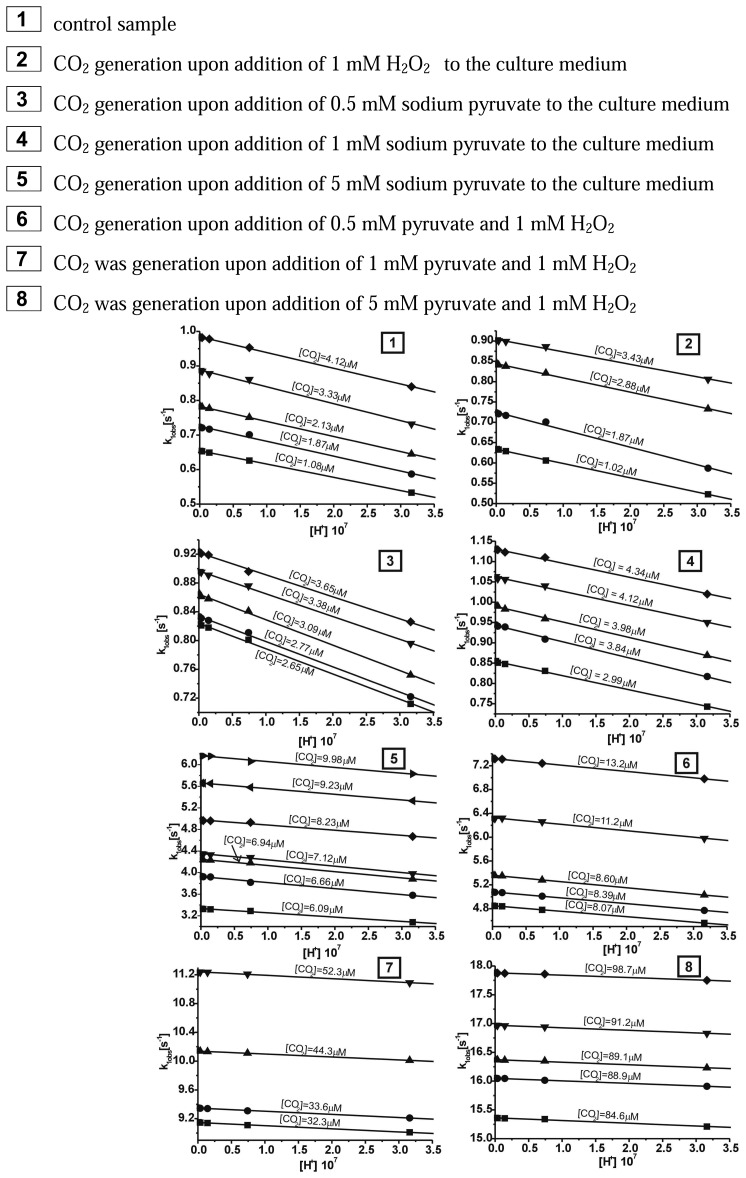
Dependence rate constants (k_1obs_) from [H^+^] for the carbon dioxide uptake by the *cis*-[Cr(C_2_O_4_)(pm)(OH_2_)_2_]^+^ ion in the T=15°C.

**Figure 3.**
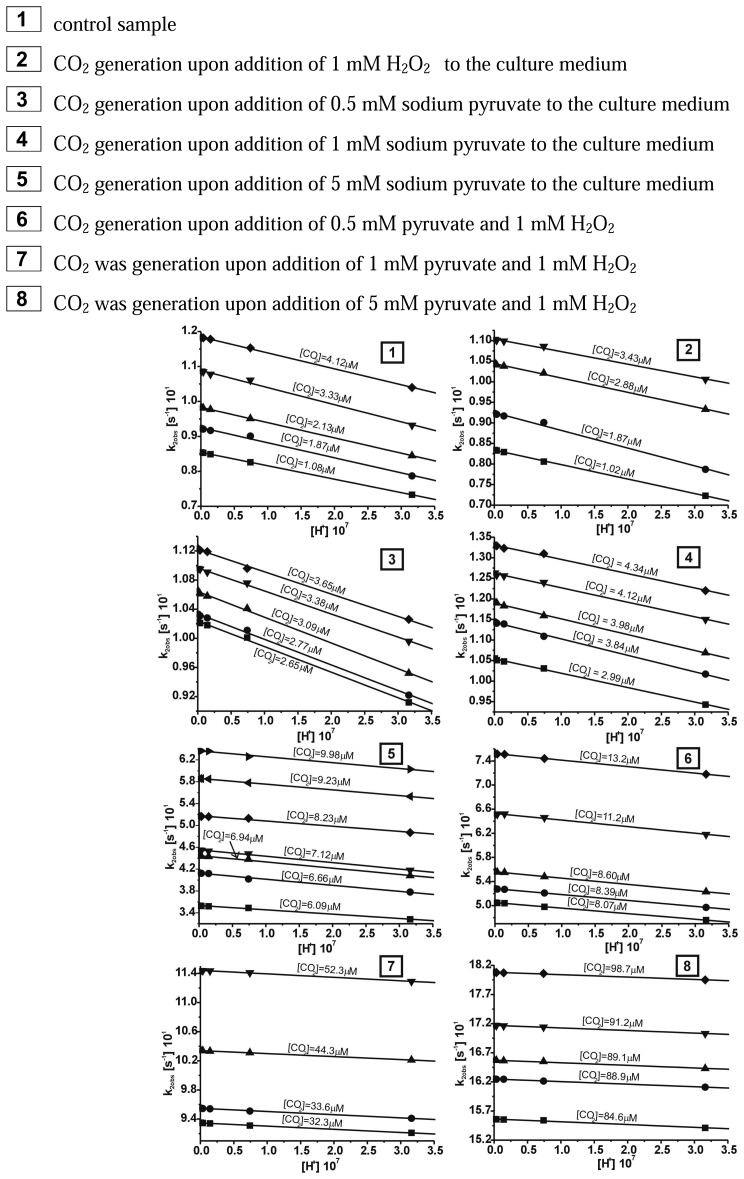
Dependence rate constants (k_2obs_) from [H^+^] for the ring closure by the *cis*-[Cr(C_2_O_4_)(pm)(OH_2_)_2_]^+^ ion in the T=15°C.

The coordination compound of Cr(III) with as bidendate ligand - pyridoxamine turned out to be successfully applied in the case of the detection of CO_2_ generated in the reaction of decarboxylation of pyruvate caused by 1 mM H_2_O_2_. The reaction between the *cis*-[Cr(C_2_O_4_)(pm)(OH_2_)_2_]^+^ ion and carbon dioxide in aqueous solutions was observed between 340 – 700 nm by using stopped-flow method. In the first step of our studies the chemical model for this reaction was adopted, which had been already described in earlier paper [26]. While carrying out the measurements of CO_2_ uptake reaction by the *cis*-[Cr(C_2_O_4_)(pm)(OH_2_)_2_]^+^ cation it was noted that for all probes studied the approximated curve decayed bi-exponentially. It should be stressed that the reaction studied proceeded in two steps. Observable rate constants k_1obs_ for the carbon dioxide uptake and k_2obs_ for the ring closure for this compound were obtained by fitting the rate data to the pseudo-first order kinetic equation for consecutive reaction model (A→B→C). The results showing the dependence [H^+^] from observable constant rates (k_obs_) are demonstrated in Figures 2 and 3, respectively.

The results of calculations for reaction studied showed that at increasing [H^+^] and T=const the observable rate constant increased for carbon dioxide uptake (k_1obs_ [s^-1^]) and the ring closure stages (k_2obs_ [s^-1^]).

**Figure 4.**
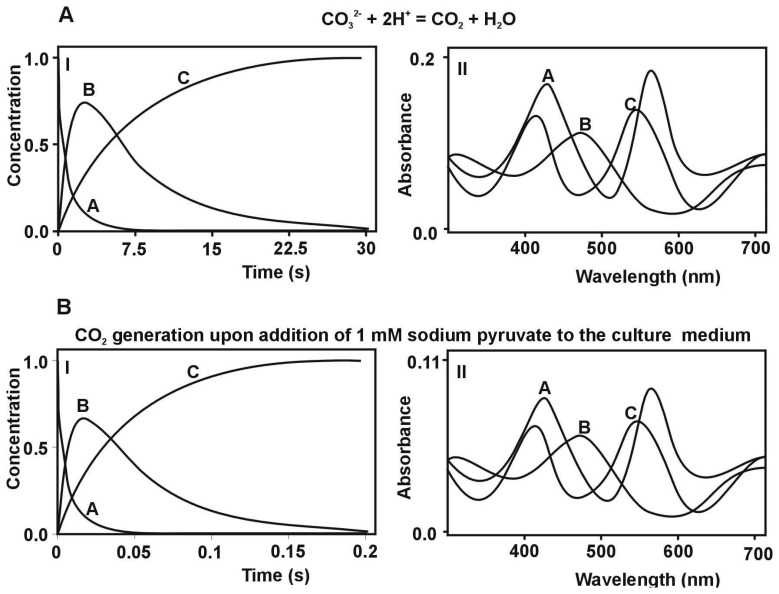
The comparison of kinetic and spectral characteristics of reactants in simple inorganic CO_2_ generating system. **(A)** (I) Curves of concentration decay and buildup of the substrate A (which is the *cis*-[Cr(C_2_O_4_)(pm)(OH_2_)_2_]^+^ ion), product C as *cis*-[Cr(C_2_O_4_)(pm)(O_2_CO)]^-^ ion, and intermediate product B. (II) Absorption spectra of the reactants A, B and C in pH = 7.13, [CO_2_] = 0.01M, T=20°C. **(B)** (I) Curves of concentration changes for reaction of CO_2_ uptake observed for the substrate A (which is the *cis*-[Cr(C_2_O_4_)(pm)(OH_2_)_2_]^+^ ion), product C as *cis*-[Cr(C_2_O_4_)(pm)(O_2_CO)]^-^ ion, and intermediate product B. (II) Absorption spectra of the reactants A, B and C in pH = 6.5, [CO_2_] = 8.23M T=20°C.

These results could be also treated as a confirmation of bistage reaction type. In the first step, an intermediate compound B is formed and subsequently converted to a final product C, characteristic for the second step. The results of global analysis, for reaction of CO_2_ uptake by the *cis*-[Cr(C_2_O_4_)(pm)(OH_2_)_2_]^+^ ion within the consecutive reaction model are presented in Figure 4.

The chemical and biological models of the reaction of CO_2_ uptake have been suggested for *cis*-[Cr(C_2_O_4_)(pm)(OH_2_)_2_]^+^ complex ion based on absorption spectra which are shown in Figure 4 (A). During the carbon dioxide uptake (where both CO_2_ was generated in chemical reaction; as well as) by *cis*-[Cr(C_2_O_4_)(pm)(OH_2_)_2_]^+^ complex ion the most significant changes of absorbance could be seen at λ = 560 nm. Furthermore, in the reaction between pyruvate and H_2_O_2_ in culture medium) It can be treated as a confirmation that the proposed chemical model fits to the biological system. This testifies about very. At the first step (carbon dioxide uptake), kinetics data were fitted by simple A→B reaction model (where B denotes moving-product). At the second step (the closure of the ring of carbonate ion), the reaction was monitored at maximum differences in molar absorptivities between the moving-products and products (B→C reaction model). It should be pointed that using two methods; namely the singular value decomposition (SVD) analysis and global analysis (GA), the same results were obtained. Next the mathematical model for CO_2_ uptake by *cis*-[Cr(C_2_O_4_)(pm)(OH_2_)_2_]^+^ was proposed. Based on the determined acidity constants, K_1_ and K_2_ and the observable rate constants, k_1obs_ the final equation was obtained:

(3) $$\frac{{\text{k}}_{1\text{obs}}({\left[{\text{H}}^{+}\right]}^{2}+{\text{K}}_{1}[{\text{H}}^{+}]+{\text{K}}_{1}{\text{K}}_{2}}{{\text{K}}_{1}[{\text{CO}}_{2}]}={\text{k}}_{1}[{\text{H}}^{+}]+{\text{K}}_{2}{\text{K}}_{2}$$

**Figure 5.**
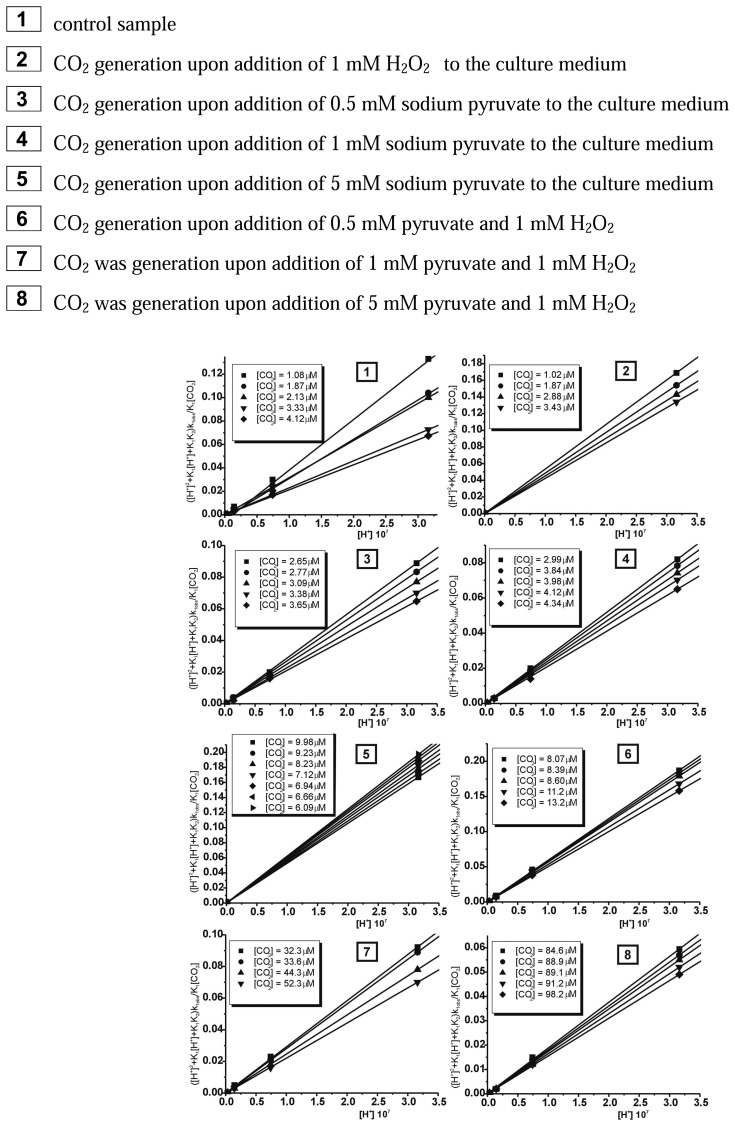
Plots of rate expression (eq. 3) for different concentration carbon dioxide uptake by *cis*-[Cr(C_2_O_4_)(pm)(H_2_O)_2_]^+^.

Then, based on the linear relationship between ([H^+^]^2^ + K_1_[H^+^] + K_1_K_2_)k_1obs_/K_1_[CO_2_] and [H^+^], rate constants k_1_[s^-1^M^-1^] and k_2_[s^-1^M^-1^] were determined for each concentration of CO_2_ (T=15°C) in the whole pH range between the measured and calculated pK_1_ and pK_2_ values. Plots of ([H^+^]^2^ + K_1_[H^+^] + K_1_K_2_)k_1obs_/K_1_[CO_2_] vs. [H^+^] yield straight lines with slopes k_1_ and intercepts of k_2_K_2_ as shown in Figure 5.

As it is evident from Figure 5, the intercepts at each temperature are negligibly small. This phenomenon is certainly not due to the inactivity of the *cis*-[Cr(C_2_O_4_)(pm)(OH)_2_]^-^ ion species, but rather to the large uncertainty of k_1obs_ at higher pH owing to the slow concurrent hydrolysis of CO_2_ at high pH [27-28]. The rate constants k_1_ [s^-1^M^-1^] and k_2_ [s^-1^M^-1^] at various concentrations of carbon dioxide for *cis*-[Cr(C_2_O_4_)(pm)(OH)_2_]^-^ ion are listed in Table 1.

The results, which are collected in Table 1, show that rate constant k_1_ [s^-1^M^-1^] (involves the reaction of CO_2_ with the monohydroxo complex) is larger than k_2_ [s^-1^M^-1^] (involves the reaction of CO_2_ with the bishydroxo species) at all concentrations of carbon dioxide.

It has been found that during the second step a final product, *cis*-[Cr(C_2_O_4_)(pm)(O_2_CO_2_)]^-^, is formed from the intermediate, *cis*-[Cr(C_2_O_4_)(pm)(O_2_COH)(OH_2_)]^0^. Since at increasing [CO_2_] and pH the rate constant k_2obs_ (Figure 3) decreases, it can be concluded, that among the three protolytic forms existing in solution, the ring closure occurs more readily in the *cis*-[Cr(C_2_O_4_)(pm)(OCO_2_H)(OH_2_)]^0^ compound. For the second step only the observable rate constant k_2obs_ was determined whereas both the kinetic equations and the acidity constant were not determined. This was due to the fact that the second step was disturbed by the hydrolysis reaction of the product, *cis*-[Cr(C_2_O_4_)(pm)(O_2_CO)]^-^ anion.

Proposed mechanism assumes that the reaction proceeds in two steps. In the first rapid step, CO_2_ is captured to *cis*-[Cr(C_2_O_4_)(pm)(OH_2_)_2_]^+^ ion and forms an intermediate product, in which the carbonate ion is linked to chromium(III) through one oxygen atom. In the second step new bond is formed between the hydrogen atom of the OH group (donor) and the oxygen atom of CO_2_ (acceptor).

Described method of CO_2_ detection allowed us to correlate the amount of released CO_2_ to culture medium with cytoprotective properties of pyruvate. Desagher *et al.* proved that extracellular pyruvate, as well as some other α - ketoacids were able to protect neurons against both exogenous and endogenous produced H_2_O_2_. They also confirmed that in scavenging activity of pyruvic acid direct reaction with oxidant – H_2_O_2_ played crucial role and it was completely independent of pyruvate's influence on energy state of cells [29]. In our experiments we also confirmed that the addition of sodium pyruvate to the culture medium protected *osteosarcoma 143B* cells in a dose-dependent manner. Moreover, we proved that the relation between the level of CO_2_ and cell survival could be useful method for the assessment of the antioxidant activities of purivic acid.

**Figure 6.**
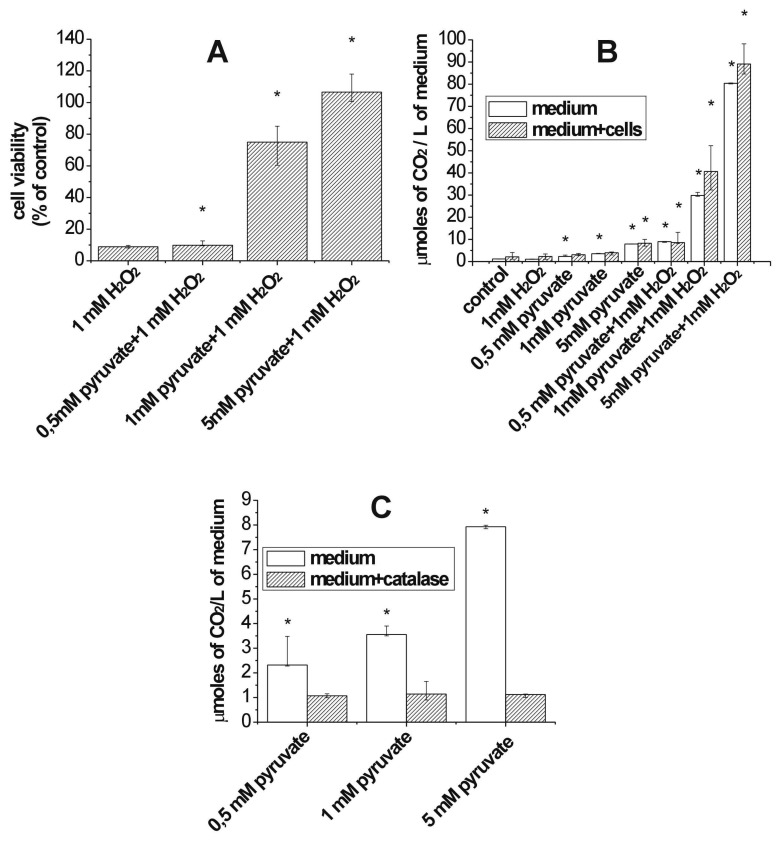
**(A)** Comparison of antioxidant protective effects of three concentration of pyruvate (0.5 mM, 1 mM and 5 mM) on viability of *Osteosarcoma 143B* cells exposed to 1 mM H_2_O_2_. The strongest protection was observed after 30 min of preincubation cells with 5 mM sodium pyruvate. The cytoprotective effect of 1 mM concentration was only partial. The lowest 0.5 mM sodium pyruvate was unable to protect against cell injury caused by H_2_O_2_. The results were analyzed by the Wilcoxon signed-rank test. The results are presented as median, 5th percentile/95th percentile. (*) P<0.05 were considered statistically significant when compared to the cell viability upon addition 1 mM H_2_O_2_ alone; **(B)** CO2 assessment in cell culture media upon addition of pyruvate and H2O2 The concentration was measured using novel stopped-flow method and specific CO2 detector - cis-[Cr(C2O4)(pm)(OH2)2]+. The results were analyzed by the Wilcoxon signed-rank test. The data are presented as median, 5th percentile/95th percentile. (*) P<0.05 were considered statistically significant when compared with respective control; **(C)** Pyruvate scavenging of endogenously produced H2O2 in cell media. The effect of catalase addition. The results of CO2 concentration upon addition pyruvate to the medium were compared with respective data obtained after preincubation with 1U/ml of catalase. The results were analyzed by the Wilcoxon signed-rank test. The data are presented as median, 5th percentile/95th percentile. (*) P<0.05 were considered statistically significant.

In our experiments we examined the capacity of sodium of pyruvate (we used three concentrations of 0.5 mM, 1 mM and 5 mM) to protect cultured *osteosarcoma* 143B cells exposed to 1 mM H_2_O_2_. The significant protection against H_2_O_2_ induced toxity was noted only for 1 mM and 5 mM concentrations of pyruvate what is in agreement with published data [30, 31]. When cells were preincubated with 0.5 mM sodium pyruvate (95% confidence intervals [CI], median 9.9%, range 9.51-12.54 of viable cells in compare to control) we observed slightly differences in cells survival in comparison to cells treated with H_2_O_2_ alone (95% CI, median 8.87%, range 8.13-9.77 of control) [Figure 6 (A)]. The level of CO_2_ increased only 4-times in comparison to control [Figure 6 (B)]. Among concentration used in our experiments the 0.5 mM sodium pyruvate was the closest to physiological concentration of endogenous pyruvate which is between 0.1 and 0.2 mM in arterial plasma [32, 33]. 1 mM sodium pyruvate was much more effective but it was still unable to protect completely *osteosarcoma* cells form injury caused by H_2_O_2_ (95% CI, median 74.99%, range 60.37-85.02 of control) [Figure 6 (A)]. It is well known that pyruvate in the milimolar concentrations reacts with H_2_O_2_ in a 1:1 stoichiometry. However, the observed ineffectiveness in protection can be explained by the reactivity of H_2_O_2_ which may react with exogenous pyruvate, as well as with crucial elements of the cells in the same time [34]. The level of CO_2_ generated when both reactants were added at 1 mM concentration increased 16-times in comparison to control [Figure 6 (B)]. The highest concentration of CO_2_ was detected for 5 mM sodium pyruvate, which was also the most effective protectant against cell injury caused by H_2_O_2_ (95% CI, median 106,61% of control, range 100.59 – 117.98) [Figure 6 (A)]. After incubation with 5 mM sodium pyruvate the CO_2_ concentration in the medium was about 36-times higher than in control [Figure 6 (B)]. This result is in a good agreement with observation that 5 mM sodium pyruvate not only completely protected cells but even induced cell proliferation [Figure 6 (A)]. The statistic analyses did not reveal significant differences between CO_2_ generation in the medium with or without cells. However, it is possible that for the slight increase in CO_2_ concentration observed in the presence of cells the metabolic transformation by pyruvate dehydrogenase is responsible. The pyruvic acid is known as an energy substrate and excess of it could have probably caused improvement in cellular metabolism and thus stimulation of cells growth [35]. The comparison of control media with and without cells indicated that for the initially 1,16 μM (95% CI, range 1,11-1,19) concentration of CO_2_ in the control medium is responsible not only cellular respiration and activity of pyruvate dehydrogenase an enzyme which transforms pyruvate to Acetyl-CoA and CO_2_[36] but also the presence of CO_2_ which comes from the 5% CO_2_ in the culture air. CO_2_ is in the equilibrium with the HCO_3_^-^ (NaHCO_3_ was a component of media) in the medium to ensure a pH value close to 7.2 necessary to proper cells growth. Furthermore, our experiments showed that after addition of pyruvate into cell culture medium one might observe evolution of CO_2_ from reactivity of pyruvate with endogenous produced H_2_O_2_ [Figure 6 (C)] Thus it is very important to note that pyruvate supplementation not only protects cells exposed to oxidative stress but also prevents artefactual response of cell culture system from unexpected stress generation from cell culture medium components [37].

## 3. Materials and Methods

### 3.1. Reagents

Dihydrochloride pyridoxamine was purchased from Sigma. The cis form of diaquapyroxamino-oxalatochromate(III) was prepared according to standard literature procedures [25]. The final products, *cis*-[Cr(C_2_O_4_)(L-L)(O_2_CO)]^-^ (where L-L denotes bidentate ligand – pyridoxamine (pm)) was synthesised by a modification of the method described in [38]. An aqueous solution of K_2_Cr_2_O_7_ (4.0 g, 8 mL) was gradually added to H_2_C_2_O_4_.2H_2_O solution (12 g, 17 mL). The precipitated crystals of *trans*-K[Cr(C_2_O_4_)_2_(OH_2_)_2_].3H_2_O were filtered off and flushed with ice-cold water and ethanol. Next, the solution of *trans*-K[Cr(C_2_O_4_)_2_(OH_2_)_2_].3H_2_O (1.96 g, 40 mL) was heated for 15 minutes (70°C – 75°C), after which its pH was adjusted to around 9. A stoichiometric quantity of pyridoxamine (5 mmol, 10 mL, pH ≈ 9) was added to this solution and then the mixture was stirred for 15 minutes, cooled and acidified with 0.5 M HClO_4_ to pH ≈ 2. The complex anion was separated by ion-exchange column chromatography (DOWEX 1 × 8 anionite). Fe(NO_3_)_3_ (0.2 M, 25 mL) and HNO_3_ (2 M, 15 mL) were added to the *cis*-[Cr(C_2_O_4_)_2_(pm)]^-^ ion solution (180 mL). After being heated for 20 minutes (318 K) the solution was left to cool. The post-reaction mixture was gradient-eluted in a chromatographic column. Next, the pH of the solution of *cis*-[Cr(C_2_O_4_)(pm)(OH_2_)_2_]^+^ (1 g, 10 mL) was adjusted to 8.5 by the portion-wise addition of aq. K_2_CO_3_ (32 mg, 10 mL). The solution was stirred for 10 minutes, then was cooled to 0°C. The composition of the product, blue crystals of *cis*-K[Cr(C_2_O_4_)(pm)(O_2_CO)], was determined by elemental analysis. The results of analytical calculations for KC_11_H_12_CrN_2_O_9_: C, 32.43; H, 2.95; N, 6.88 were in good agreement with those obtained from the elemental analysis: C, 32.44.; H, 3.00; N, 6.87. Using analytical techniques the molar ratios for components of the complex *cis*-[Cr(C_2_O_4_)(pm)(O_2_CO)]^-^ ion were obtained: Cr(III) : C_2_O_4_^2-^ : pm : CO_3_^2-^ = 1 : 1 : 1 : 1. The complex ion, *cis*-[Cr(C_2_O_4_)(pm)(O_2_CO)]^-^, was decomposed into its components in the presence of the Cr(II)_(aq)_ ion in an argon atmosphere [39]. Chromium(III) and pyridoxamine were quantitatively characterised spectrophotometrically [40]. To identify CO_3_^2-^ anion the potentiometry titration method was used. A standard solution of HCl (0.102 M) in the presence of 1% aq. methyl orange [41] was used in each titration.

### 3.2. Cell culture

The *osteosarcoma 143B* cell line (ATCC-8303) was cultured at 37°C in a humidified atmosphere with 5% CO_2_ in Dulbecco's Modified Eagle's Medium supplemented with 10% heat-inactivated fetal bovine serum and penicillin (100 μg/ml) / streptomycin (100 μg/ml) without sodium pyruvate (Sigma Chemicals Co., St. Louis, MO, U.S.A).

### 3.3. Cell treatment

The cells passed by standard methods of trypsinization using 0.25% trypsin and 0.02% EDTA solution, cultured under conditions described above for 24 hours before replacing with experimental medium containing different concentrations of sodium pyruvate (0.5 mM, 1 mM and 5 mM). After 30 minutes of preincubation 1 mM H_2_O_2_ (Sigma Chemicals, U.S.A) was added. Cells were incubated for next 24 hours before the level of CO_2_ in the medium, as well as cell viability were measured.

### 3.4. Preincubation of medium with catalase

To exclude possible involvement of culture medium as a source of H_2_O_2_ which may cause decarboxylation of pyruvate 1 unit/ml of catalase (Sigma Chemicals, U.S.A) was added to the medium for 30 minutes before addition of sodium puryvate. Then, after 24 hours of incubation the CO_2_ was measured.

### 3.5. Cell viability: MTT assay

The cytoprotective properties of pyruvate were determined using MTT assay. Briefly, the *osteosarcoma* 143B cells were seeded onto 96-well plates at the density of 4 × 10^3^ per well and cultured for 24 hours, then cells were treated with sodium pyruvate and H_2_O_2_. After 24 h 0,5 mg/ml of 3-[4,5-dimethylthiazol-2-yl]-2,5-diphenyltetrazolium bromide (MTT) was added. The plates were incubated for 4 h and supernatant was removed after centrifugation (700 xg for 10 min). Finally, 100 μL of DMSO (Sigma Chemicals, U.S.A) was added. The absorbance was recorded using an Jupiter ELISA reader (ASYS Hitech) at 550 nm wavelength with references at wavelength of 630 nm and the cells survival was calculated. Percentage viability was defined as a 100% times the ratio of absorbance in the samples to the average absorbance in the control (untreated cells).

### 3.6. Kinetic measurements and simulation

Two buffer solutions were used: 0.2 M MES [2-(4-morpholino)ethanesulfonic acid] and 0.2 M TRIS [tris(hydroxymethyl)-aminomethane], both prepared by dissolving appropriate samples in MiliQ water. pH was measured by using a CX 731 pH-meter (reading accuracy of 0.01 pH unit) and a combined electrode manufactured by Hanna. Solutions of the studied complex were prepared by mixing 0.5 mL of the *cis*-[Cr(C_2_O_4_)(L-L)(OH_2_)_2_]^+^ (C = 10^-3^ M) with 2 mL of 0.2 M MES or TRIS and 2 mLof 2 M NaClO_4_ solutions. The reaction studied was investigated over the pH range 6.50 < pH < 9.12 and at T = 15° C. The cells were seeded onto 100-mm culture plates at the density of 1.10^6^ per plate and incubated and treated as described before, than the culture medium was collected and used for CO_2_ concentration analysis by using stopped-flow spectrophotometry method.

### 3.7. Instrumentation

Spectral measurements were recorded in the UV-Vis region using a Perkin-Elmer Lambda 18 Instrument with the scan accuracy of 1 nm and 1 nm slit width at a scanning rate of 120.00 nm min^-1^. The pK_1_ and pK_2_ values in the ground state were computed by using Origin 6.0 program, based on absorbance variations at a selected wavelength and by using non-linear least squares method according to the equation described in [42]. Kinetic measurements were carried out using a stopped-flow technique and an Applied Photophysics SX-17MV spectrophotometer. The observable rate constants were computed using a “Glint” program based on global analysis [43-46].

### 3.8. Statistical Analysis

Data were computed using Statistica 7.1 program (Statsoft, Poland). Parametric and non-parametric distribution was assessed by Shapiro-Wilk test. The analysis was based on non-parametric statistic Wilcoxon test signed-rank as indicated by data distribution.

## 4. Conclusion

In this paper we described a new method of carbon dioxide detection in the physiological cell culture medium. Moreover, we have presented usefulness of our method, which is based on the interaction of CO_2_ with the coordination compound of *cis*-[Cr(C_2_O_4_)(pm)(OH_2_)_2_]^+^ ion for the CO_2_ detection in biological samples. Furthermore, the kinetic studies of carbon dioxide uptake by *cis*-[Cr(C_2_O_4_)(pm)(OH_2_)_2_]^+^ ion using the stopped-flow method enabled us to conclude that the reaction studied proceeded in two steps, namely carbon dioxide uptake (first step) and the closure of the ring of carbonate ion (the second step). The presented method above seems also to be handy tool to analyse scavenging reaction of H_2_O_2_ by pyruvate in biological samples. The measurement of CO_2_ concentration can be used not only to analyse chemical reaction but it can be also a marker of pyruvate's protection efficiency against oxidative stress.

Our results provided arguments for usefulness of pyruvate application for cell culture studies where culture media could produce artefectually significant levels of H_2_O_2_ before treatment of cells. On the other hand endogenous and exogenous source of H_2_O_2_ being implicated in cytotoxity in variety of human diseases can be safety prevented by pyruvate. The efficiency of this scavenger was clearly demonstrated by novel application of CO_2_ molecular biosensor.

## Figures and Tables

**Table 1.** Rate parameters for the carbon dioxide uptake reaction by *cis*-[Cr(C_2_O_4_)(pm)(OH_2_)_2_]^+^ Ion in T=20°C.

**CO_2_ [M]**	**k_1_ [ms^-1^ mM^-1^]**	**k_2_ [s^-1^ M^-1^]**
CO_2_ was generated control sample		

1.08	5.61E-1	7.88E-1
1.87	5.68E-1	7.89E-1
2.13	5.7E-1	7.91E-1
3.33	5.94E-1	8.07E-1
4.12	6.03E-1	8.13E-1

CO_2_ was generated upon addition of 1 mM H_2_O_2_ to the culture medium		

1.02	5.61E-1	7.82E-1
1.87	5.68E-1	7.89E-1
2.88	5.88E-1	8.03E-1
3.43	5.95E-1	8.08E-1

CO_2_ was generated upon addition of 0.5 mM sodium pyruvate to the culture medium		

2.65	5.81E-1	7.95E-1
2.77	5.87E-1	7.98E-1
3.09	5.92E-1	8.06E-1
3.38	5.94E-1	8.07E-1
3.65	5.96E-1	8.09E-1

CO_2_ was generated upon addition of 1 mM sodium pyruvate to the culture medium		

2.99	5.91E-1	8.05E-1
3.84	5.98E-1	8.10E-1
3.98	6.01E-1	8.11E-1
4.12	6.03E-1	8.13E-1
4.34	6.05E-1	8.15E-1
**CO_2_ [M]**	**k_1_ [ms^-1^ mM^-1^]**	**k_2_ [ms^-1^ mM^-1^]**

CO_2_ was generated upon addition of 5 mM sodium pyruvate to the culture medium		

6.09	6.07E-1	8.18E-1
6.66	6.12E-1	8.21E-1
6.94	6.14E-1	8.23E-1
7.12	6.17E-1	8.25E-1
8.23	6.28E-1	8.29E-1
9.23	6.35E-1	8.55E-1
9.98	6.41E-1	8.63E-1

CO_2_ was generated upon addition of 0.5 mM pyruvate and 1 mM H_2_O_2_		

8.07	6.23E-1	8.27E-1
8.39	6.29E-1	8.32E-1
8.60	6.31E-1	8.47E-1
11.2	6.53E-1	8.91E-1
13.2	7.22E-1	9.31E-1

CO_2_ was generated upon addition of 1 mM pyruvate and 1 mM H_2_O_2_		

32.3	8.73E-1	1.09
33.6	9.02E-1	1.11
44.3	1.01	1.26
52.3	1.09	1.31

CO_2_ was generated upon addition of 5 mM pyruvate and 1 mM H_2_O_2_		

84.6	1.41	1.67
88.9	1.43	1.68
89.1	1.44	1.71
91.2	1.48	1.74
98.2	1.54	1.81

The error in k_1_[ms^-1^mM^-1^] and k_2_[ms^-1^mM^-1^] oscillates from 0.8% to 2.8%.
